# Calcium Hydroxylapatite-Induced Inflammatory Facial Edema and Induration Due to Hashimoto’s Thyroiditis

**DOI:** 10.7759/cureus.40947

**Published:** 2023-06-25

**Authors:** Kritin K Verma, Katherine A Edwards, Daniel P Friedmann

**Affiliations:** 1 School of Medicine, Texas Tech University Health Sciences Center, Lubbock, USA; 2 Dermatology, Henry Ford Health System, Detroit, USA; 3 Dermatology, Westlake Dermatology Clinical Research Center/Westlake Dermatology & Cosmetic Surgery, Austin, USA

**Keywords:** levothyroxine, hashimoto's thyroiditis, filler nodule, filler complication, calcium hydroxylapatite

## Abstract

We describe a patient who experienced a diffuse, treatment-refractory facial inflammatory reaction following the injection of calcium hydroxylapatite with lidocaine. The reaction was attributed to undiagnosed Hashimoto's thyroiditis. Exogenous thyroid hormone replacement therapy rapidly resolved the facial inflammation associated with this type of autoimmune hypothyroidism.

## Introduction

Calcium hydroxylapatite (CaHA) with or without integral 0.3% lidocaine hydrochloride (Radiesse® and Radiesse® +, respectively, Merz North America, Inc., Raleigh, NC) is a biostimulatory soft tissue filler approved by the U.S. Food & Drug Administration in 2006 for the correction of moderate-to-severe wrinkles and folds of the face [1]. There have been prior reports of inflammatory complications associated with CaHA, including nodules and alopecia secondary to foreign-body reactions [2,3]. We present here a patient who experienced a diffuse inflammatory reaction to CaHA with integral lidocaine due to undiagnosed Hashimoto’s thyroiditis.

## Case presentation

**Figure 1:**
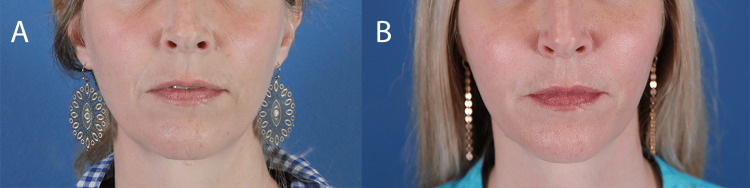
Facial edema from calcium hydroxylapatite Before (A) and 3 weeks following (B) subdermal injection with a 1.5 mL syringe of calcium hydroxylapatite with integral lidocaine to the temples, malar and buccal cheeks, nasolabial folds, and marionette lines. Diffuse edema is evident in treated areas of the face.

A healthy 43-year-old woman (Figure 1A) was treated with 1 syringe (1.5 mL) of CaHA with integral lidocaine. Subdermal injection was performed into the temples, malar and buccal cheeks, nasolabial folds, and marionette lines using a 25G blunt-tipped cannula. The patient had previously been treated with CaHA, poly-L-lactic acid, and hyaluronic acid fillers in these same facial locations without complications. Three weeks post-injection, the patient complained of progressive swelling and firmness of her cheeks of 3 days duration. Physical examination revealed diffuse induration and warmth in treated areas (Figure 1B), without evidence of pain, tenderness, or intense erythema that would indicate cellulitis. The patient denied fever or recent illnesses. She was started on minocycline 100 mg capsules po BID for 2 weeks and prednisone 10 mg po Qdaily for 3 days for a suspected foreign body reaction.

Two weeks later, the patient reported no improvement, despite completing the course of minocycline and prednisone. On examination, there was persistent painless, non-fluctuant subcutaneous edema, induration, and warmth that was greatest in the bilateral malar and buccal cheeks, but also present in the nasolabial folds, temple, and marionette line areas. The patient was prescribed furosemide 20 mg po Qdaily for 2 days to help reduce the edema and doxycycline 75 mg po Qdaily for 1 month as a continued anti-inflammatory, but neither led to significant improvement. However, the patient was shortly later diagnosed with Hashimoto’s thyroiditis by her primary care physician and started on levothyroxine po Qdaily (titrated to a daily dose of 100 mcg), leading to complete resolution of her signs and symptoms within 7-10 days of starting the exogenous thyroid hormone.

## Discussion

CaHA is a well-tolerated, biostimulatory, and biodegradable soft tissue filler. Adverse events from CaHA (e.g., erythema, edema, pain, tenderness, induration, and ecchymosis) are primarily mild, injection-related, and transient, resolving within hours to days following treatment [1]. Subacute-onset (2 weeks to 1 year) or late-onset (>1 year) filler-associated nodules can be either non-inflammatory or inflammatory [4]. The former are asymptomatic and may be due to overcorrection, overly superficial placement, or product migration. Inflammatory nodules, on the other hand, can exhibit pain, tenderness, warmth, erythema, edema, and induration. While localized painful lesions can develop due to poor aseptic technique (subacute) or biofilm formation (late), diffuse lower-grade inflammation may be of infectious or autoimmune etiology [4]. Given the mild diffuse inflammatory reaction to CaHA in our patient, an autoimmune-mediated foreign-body granulomatous reaction, caused by macrophage activation and subsequent T-cell response, is the likely explanation. Not surprisingly, it is thought that defects in regulatory T-cells and increased activation of follicular helper T-cells play a major role in the pathogenesis of Hashimoto’s thyroiditis [5].

Due to the potential inflammatory complications of CaHA, its use should be avoided in patients with pre-existing uncontrolled autoimmune disease. Acute onset autoimmune disease should likewise be considered in patients who develop prolonged inflammatory edema and induration within several weeks of CaHA injection.

## Conclusions

As seen in this case, an autoimmune diagnosis should be ruled out in patients with acute-onset inflammation following injection of CaHA. Patients with a pre-existing diagnosis of uncontrolled autoimmune disease are best treated with hyaluronic acid fillers instead of CaHA. Inflammatory reactions to CaHA associated with autoimmune hypothyroidism may rapidly respond to exogenous thyroid hormone replacement therapy.
